# Immunohistochemical Characterization of the Androgen Receptor in Breast Cancer and Its Relationship with Breast Cancer Subtypes

**DOI:** 10.3390/medsci14040479

**Published:** 2026-08-13

**Authors:** María Luisa Sánchez-Ferrer, Alexandra Esteban Pedreño, Julián J. Gonzalo-Arense, Inmaculada Ruiz Boluda, Micaela Llamas Sarriá, Jose Luis Alonso Romero, Domingo Sánchez Martínez, Carlos Manuel Martínez-Cáceres, Jaime Mendiola, Alberto M. Torres Cantero

**Affiliations:** 1 School of Medicine, University of Murcia, 30120 Murcia, Spain; alexandraesteban11@gmail.com (A.E.P.); julianjesus.arense@um.es (J.J.G.-A.); inmab96@gmail.com (I.R.B.); mickymic96@gmail.com (M.L.S.); josel.alonso2@carm.es (J.L.A.R.); dosanz10@gmail.com (D.S.M.); jaime.mendiola@um.es (J.M.); amtorres@um.es (A.M.T.C.); 2 Department of Obstetrics & Gynecology, “Virgen de la Arrixaca” University Clinical Hospital, 30120 Murcia, Spain; 3 Biomedical Research Institute of Murcia (IMIB-Arrixaca), University Clinical Hospital “Virgen de la Arrixaca”, Pathology Platform, 30120 Murcia, Spain; carlos.martinez@imib.es; 4 Division of Preventive Medicine and Public Health, Department of Public Health Sciences, 30120 Murcia, Spain; 5 Department of Oncology, “Virgen de la Arrixaca” University Clinical Hospital, 30120 Murcia, Spain

**Keywords:** breast cancer, androgen receptor, immunohistochemistry, molecular subtypes, Ki-67

## Abstract

Background/Objectives: Breast cancer is the most frequent malignant neoplasm in women and presents marked biological heterogeneity. The androgen receptor (AR) has emerged as a biomarker with important prognostic and therapeutic implications, its effect varying according to the molecular subtype. The objective of this study was to analyze AR expression in breast carcinoma samples and its relationship with the different molecular subtypes and clinicopathological variables. Methods: An observational, descriptive, cross-sectional, and prospective study was conducted based on the immunohistochemical analysis of 215 formalin-fixed, paraffin-embedded breast carcinoma samples. AR expression was digitally evaluated as the percentage of positive tumor cells after incubation with an anti-AR monoclonal antibody. Results: A high frequency of AR expression was demonstrated in the cohort, with a median of 53.3%. There were statistically significant differences between molecular subtypes (*p* < 0.001), detecting greater expression in luminal tumors and markedly low levels in triple-negative breast cancer (TNBC) (median 0.41%). A significant negative correlation was evidenced between AR expression and the Ki-67 proliferation index (ρ = −0.272; *p* < 0.001), both in the overall sample and in the TNBC subgroup. Conclusions: The androgen receptor is associated with specific molecular subtypes and lower tumor proliferation, suggesting a less aggressive phenotype and supporting its role as a biological biomarker and potential therapeutic target in the management of breast cancer.

## 1. Introduction

Breast cancer constitutes the most frequent malignant neoplasm in women worldwide and one of the leading causes of female oncological mortality. Molecular classification based on estrogen receptors (ERs), progesterone receptors (PRs), and human epidermal growth factor receptor 2 (HER2) has allowed the design of targeted therapies. However, the treatment response remains heterogeneous due to the underlying complexity of the disease, especially in tumors such as triple-negative breast cancer (TNBC), which continue to pose a clinical challenge as they lack classical therapeutic targets.

In this context, the androgen receptor (AR), belonging to the steroid nuclear receptor family, has emerged as a valuable predictive and prognostic biomarker. The androgen receptor (AR) is a ligand-dependent transcription factor belonging to the nuclear steroid receptor superfamily. Following androgen binding, AR translocates to the nucleus and regulates the expression of genes involved in cell proliferation, differentiation, apoptosis, and metabolic pathways. Beyond its classical genomic activity, AR also activates non-genomic signaling cascades, including PI3K/AKT, MAPK, and Wnt/β-catenin pathways, which may influence breast cancer progression and therapeutic resistance. Recent evidence suggests that the biological role of AR is highly context-dependent and determined by interactions with estrogen receptor (ER) [1], HER2 signaling, and the tumor microenvironment. In ER-positive tumors, AR may antagonize estrogen-mediated transcription, whereas in ER-negative disease, AR can function as an oncogenic driver through alternative molecular pathways [2,3].

Recent scientific literature reports that AR is expressed in 60% to 90% of breast cancer cases [4,5]. However, AR function is intimately linked to the context of the molecular subtype. In luminal tumors (ER+), co-expression is very high, and AR frequently acts competitively with ER, exerting an antiproliferative protective effect [6]. Conversely, in the absence of ER (such as in TNBC), AR can promote tumor growth, associating with phenotypes that could respond favorably to androgen antagonists such as bicalutamide or enzalutamide [7].

The interaction between androgen receptor (AR) signaling and estrogen receptor (ER) pathways represents a key mechanism underlying its biological effects in breast cancer. Experimental studies have demonstrated that AR can interfere with ER-mediated transcription by competing for DNA-binding elements and transcriptional coactivators, resulting in attenuation of estrogen-driven proliferation. This regulatory interaction may contribute to the more favorable clinical behavior observed in AR-positive luminal tumors [8,9].

AR activation has been shown to reprogram gene expression, repressing cell cycle-related genes while promoting differentiation-associated transcriptional profiles.

In contrast, in ER-negative tumors, AR may act as an oncogenic driver through activation of alternative signaling pathways. These include interactions with HER2 signaling and the activation of Wnt/β-catenin cascades, which are known to promote tumor growth and resistance to therapy [2,3].

This dual role has led to the recognition of AR as a context-dependent biomarker. In particular, the luminal androgen receptor (LAR) subtype of triple-negative breast cancer (TNBC) has been described as a distinct entity characterized by androgen-signaling dependency despite the absence of ER and PR expression [10,11]. These tumors may represent a clinically relevant subgroup with potential sensitivity to targeted anti-androgen therapies [12,13]. In addition, a high expression of androgen in ER-positive breast cancer can predict resistance to therapies like tamoxifen and identify alternative targets for combination treatments. Contrarily, the antitumor effect of aromatase inhibitors in postmenopausal patients relies partially on androgen precursors that interact with the AR. In this setting, AR signaling actually helps inhibit tumor proliferation.

Taken together, these findings highlight the importance of considering AR within its molecular context, rather than as an isolated biomarker, when evaluating its role in breast cancer biology and clinical management.

The general objective of this study is to describe AR expression in a cohort of patients with breast carcinoma and to analyze how it is distributed based on molecular subtypes (Luminal A, Luminal B, HER2+, and triple-negative). Furthermore, its correlation with proliferative parameters, specifically Ki-67, is analyzed to provide evidence on its viability as a new therapeutic target in patients with a poor response to conventional treatment.

## 2. Materials and Methods

An observational, descriptive, cross-sectional, and prospective study was conducted using 215 formalin-fixed, paraffin-embedded (FFPE) breast carcinoma tissue samples with a confirmed histological diagnosis. Samples were obtained from the archive of the Department of Pathology at Hospital Clínico Universitario Virgen de la Arrixaca (Murcia, Spain). This study was carried out within the framework of research project PI22/00337.

For immunohistochemical analysis, 3 µm tissue sections were prepared. Breast cancer cases were classified according to current clinical practice guidelines into molecular subtypes based on estrogen receptor (ER), progesterone receptor (PR), Ki-67 index, and HER2 status: Luminal A (ER+/high PR, HER2–, Ki-67 < 20%, ~40% prevalence); Luminal B (ER+/low PR, HER2–, Ki-67 > 20%, ~20%); Luminal HER2-positive (ER+ and/or PR+, HER2+, variable Ki-67); Pure HER2-overexpressed (ER–/PR–, HER2+); and Triple-negative (ER–/PR–, HER2–, high Ki-67, ~15%) [14].

**Figure 1:**
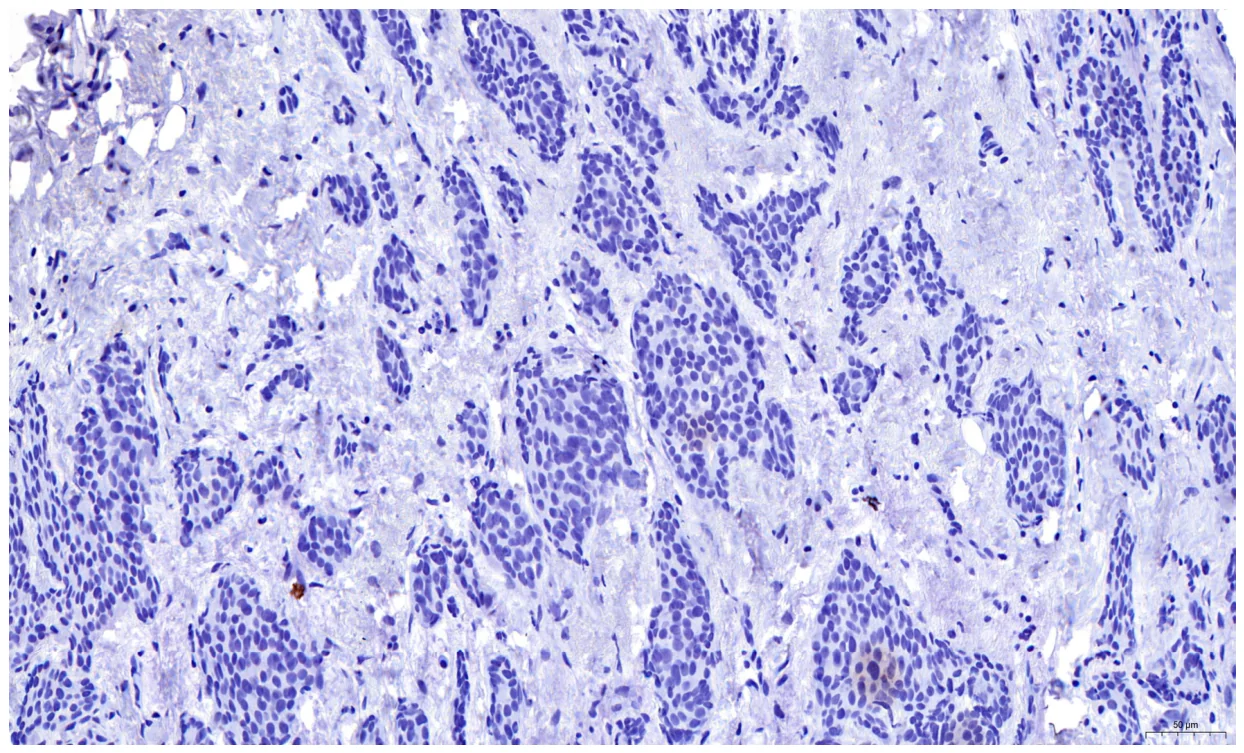
Absence of androgen receptor (AR) expression in breast carcinoma tumor cells. No specific nuclear staining is observed. Immunohistochemical staining. Original magnification 250×.

**Figure 2:**
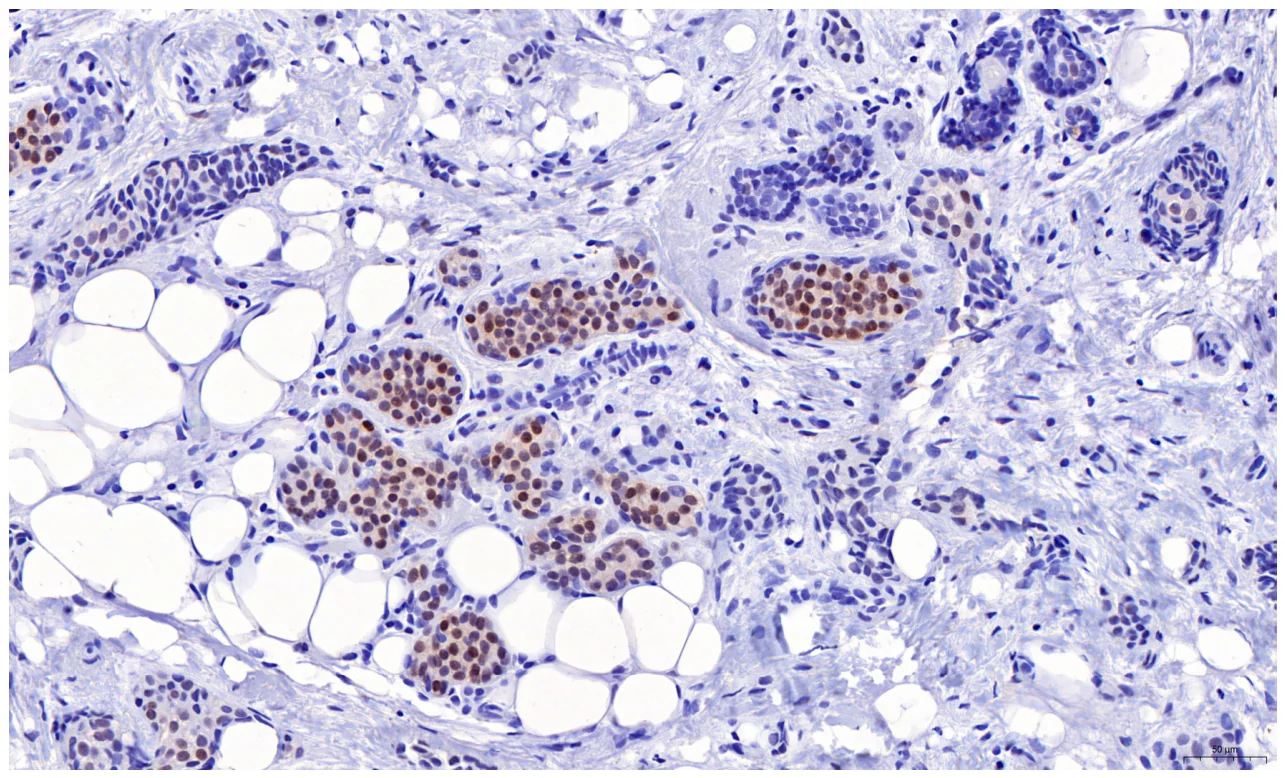
Intermediate expression of the androgen receptor (AR) in tumor cells, with positive nuclear staining (brown colour) in a moderate percentage of cells. Immunohistochemical staining. Original magnification 250×.

**Figure 3:**
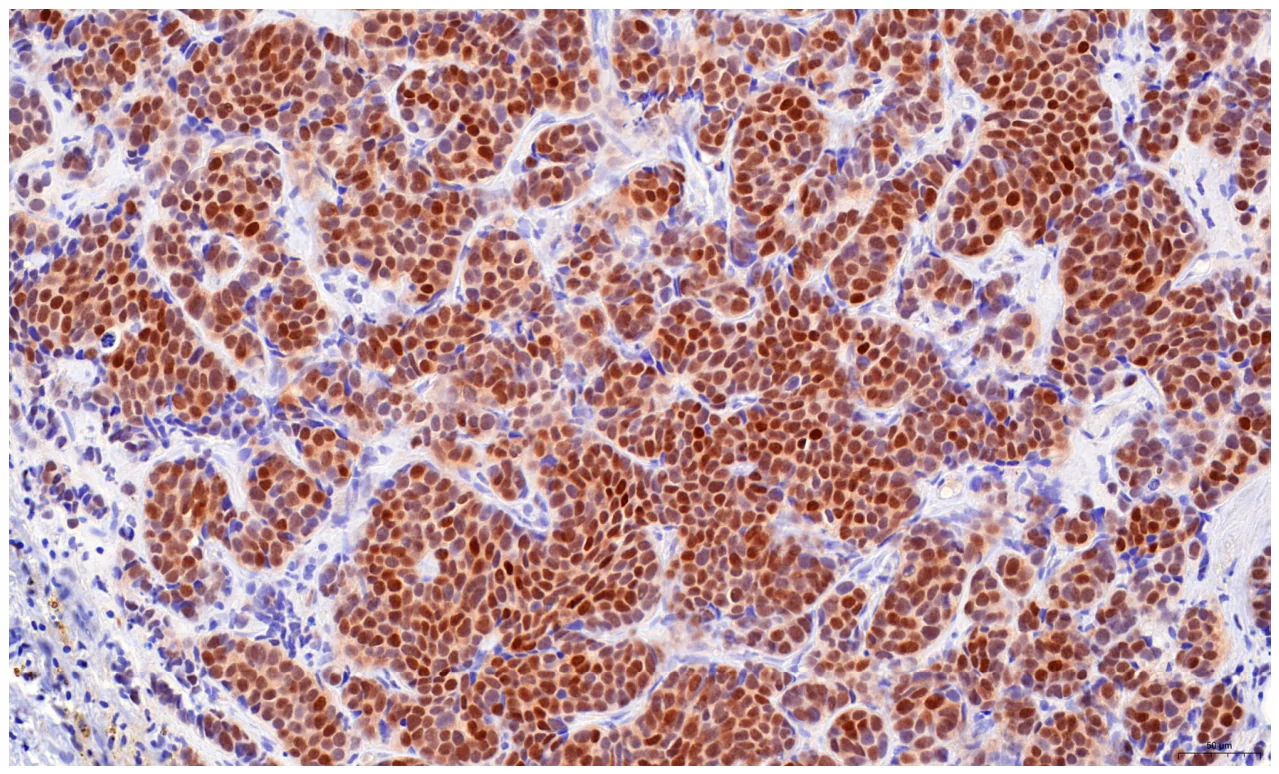
High expression of the androgen receptor (AR), with intense nuclear staining (brown colour) in most tumor cells. Immunohistochemical staining. Original magnification 250×.

To perform the AR analysis a monoclonal rabbit anti-androgen receptor (clone EPR1535(2) (catalog number ab133273) (Abcam, Oxford, UK) was employed. This antibody has been validated by the manufacturer for its use in formalin-fixed and paraffin-embedded tissues from human, mouse and rat specimens (https://www.abcam.com/en-us/products/primary-antibodies/androgen-receptor-antibody-epr15352-ab133273; accessed on 25 July 2026), and has been employed in numerous scientific publications. To verify the specificity of the immunohistochemical procedure, a positive control (human testis) was employed The same tissue without primary antibody was also used as a negative control. Androgen receptor (AR) expression was evaluated using an indirect polymer-based peroxidase system (EnVision Flex, Agilent-Dako) following heat-induced antigen retrieval. Samples were incubated with a monoclonal anti-AR antibody (Abcam, dilution 1:500), and staining was visualized using 3,3′-diaminobenzidine (DAB), followed by hematoxylin counterstaining. Digital image analysis was performed using QuPath software version 0.6.0 (Figure 1, Figure 2 and Figure 3 and Figure S1). For AR digital analysis, we employed QPATH version. 0.6.0 (build 26 June 2025). Tumors were previously selected by specialized oncologists from a cohort of diagnosed formalin-fixed and paraffin-embedded PAAF biopsies from the central archives of the pathology department of the HUCVA. All quantitative analyses were performed by investigators who were blinded to annotations of the examined slides (AEP and CMM). All specimens were anonymized to ensure blind analysis. Samples with insufficient tumor tissue were discarded from the analysis. One CNB AR-stained section for each sample was analyzed. AR expression was quantified as the percentage of tumor cells showing positive nuclear staining relative to the total number of tumor cells. Quantification was performed on manually selected regions of interest (ROIs) containing exclusively epithelial tumor cells, excluding peritumoral stroma, tissue artifacts, and non-representative areas. This approach was adopted because the objective of the analysis was to determine the proportion of AR-positive tumor cells. Furthermore, due to the nature of core-needle biopsy-derived samples, tumor cell nests showed a heterogeneous spatial distribution throughout the sections, making whole-section analysis inappropriate. All representative epithelial tumor cell areas within each core were included in the selected ROIs. On average, a total ROI area of 1,295,003 µm^2^ per sample was analyzed, comprising a mean of 9274 tumor cells.

The CNB samples included in this study had been previously diagnosed during routine clinical practice, and the Ki-67 proliferation index had already been determined according to the institutional diagnostic protocol by using a mouse monoclonal antibody (clone MIB-1, ready-to-use, cat. NºGA626) (Agilent-Dako Technologies, Madrid, Spain) in a fully automated IHC platform (Dako Omnis, Agilent Tech., Santa Clara, CA 95051, USA) following the manufacturer’s recommendations. The Ki-67 proliferation index was calculated as the percentage of positive-stained nuclei among at least 500 invasive tumor epithelial cells counted within the hot spot area showing the highest proliferative activity.

Statistical analyses were performed using SPSS software, Version 25.0. As the variables did not follow a normal distribution, non-parametric tests were applied, including Spearman’s rank correlation coefficient, the Mann–Whitney U test, and the Kruskal–Wallis test. Statistical significance was established at *p* < 0.05. To assess the association between AR expression and tumor proliferative activity, the percentage of AR-positive tumor cell nuclei was correlated with the percentage of Ki-67-positive tumor cells using Spearman’s rank correlation coefficient (ρ), given the non-parametric distribution of the variables.

The study was approved by the Biosafety Committee of the University of Murcia (Approval ID: 729/2025). Ethical approval was subsequently ratified by the Research Ethics Committee (Comisión de Ética de Investigación, CEI) of the University of Murcia (Approval Code: M10/2025/583R; 12 November 2025) and authorized by the Research Evaluation Committee of Health Area I (Murcia, Spain). All samples and associated data were anonymized prior to analysis, and all applicable ethical and biosafety regulations were strictly followed.

## 3. Results

A total of 215 breast carcinoma samples were included in the study. Complete clinicopathological and immunohistochemical data were available for the variables analyzed (Table 1).

### 3.1. Global Androgen Receptor Expression

Androgen receptor (AR) expression showed considerable variability across the cohort, ranging from 0.02% to 93.74% (Figure 1, Figure 2 and Figure 3). The median AR expression was 53.3% (interquartile range (IQR: 14.6–77.4), reflecting a heterogeneous distribution among breast cancer samples (Table 2).

Using a positivity cutoff of 1%, AR expression was detected in 189 cases (87.9%). When a more restrictive cutoff of 10% was applied, 167 tumors (77.7%) remained classified as AR-positive (Table 3).

### 3.2. Expression According to Molecular Subtype

Significant differences in androgen receptor (AR) expression were observed among the different molecular subtypes of breast cancer (*p* < 0.001).

Luminal tumors (Luminal A and Luminal B) exhibited the highest levels of AR expression, with median values above 50%. HER2-positive tumors showed intermediate expression levels, whereas triple-negative breast cancer (TNBC) displayed the lowest AR expression, with values concentrated at the lower end of the distribution (Table 4).

### 3.3. Association Between AR Expression and Biological Variables

A significant inverse correlation was observed between androgen receptor (AR) expression and the Ki-67 proliferation index (ρ = −0.272, *p* < 0.001), indicating that tumors with higher proliferative activity tended to exhibit lower AR expression.

In contrast, AR expression showed significant positive correlations with estrogen receptor (ER) and progesterone receptor (PR) expression. No significant association was identified between AR expression and HER2 status (Table 5).

### 3.4. Variable Association Between AR Expression and Clinicopathological Variables

A significant association was observed between androgen receptor (AR) expression and histological grade (*p* < 0.001). Well-differentiated and moderately differentiated tumors exhibited higher levels of AR expression, whereas poorly differentiated tumors showed substantially lower expression levels.

In contrast, no significant differences in AR expression were identified according to TNM clinical stage (*p* = 0.214) (Table 6).

### 3.5. Triple-Negative Breast Cancer Subgroup Analysis

Thirty-one tumors were classified as triple-negative breast cancer (TNBC). Overall, AR expression was low within this subgroup, with a median value of 0.41% (IQR: 0.08–6.67).

A significant inverse correlation was identified between AR expression and Ki-67 (ρ = −0.369, *p* = 0.041), suggesting that tumors with higher proliferative activity tended to exhibit lower AR expression levels (Table 7).

## 4. Discussion

This study evaluated androgen receptor (AR) expression in a cohort of 215 breast cancer samples and examined its association with molecular subtypes and clinicopathological characteristics. AR expression was detected in a high proportion of tumors, although substantial heterogeneity was observed across molecular subtypes. The main findings were the higher AR expression observed in luminal tumors, the markedly lower expression in triple-negative breast cancer (TNBC), and the inverse association between AR expression and Ki-67 proliferation index. AR expression was also associated with histological grade but not with TNM stage.

The overall frequency of AR expression observed in our cohort is consistent with previous studies reporting AR positivity in 60–90% of breast cancers [4,5]. However, variability in immunohistochemical methodologies, scoring systems, and positivity thresholds may contribute to differences between studies. Therefore, AR expression by immunohistochemical should be interpreted in the context of the specific cutoff applied. In the present study, two commonly reported thresholds (≥1% and ≥10%) were considered, allowing our findings to be interpreted alongside the methodological heterogeneity of the existing literature.

One of the main findings was the significant association between AR expression and molecular subtype. Luminal tumors exhibited the highest levels of AR expression, whereas TNBC displayed markedly lower expression. These findings are consistent with previous reports describing frequent AR expression in hormone receptor-positive breast cancer [6]. The association between AR expression and molecular subtypes supports the biological relevance of AR across breast cancer subtypes, although functional AR activity cannot be inferred from immunohistochemical expression alone.

A significant inverse association was also observed between AR expression and Ki-67 proliferation index, both in the overall cohort and in the TNBC subgroup. Tumors with higher AR expression tended to exhibit lower proliferative activity, consistent with previous observations suggesting an association between AR expression and more differentiated, less proliferative tumor phenotypes [7]. Experimental studies have suggested that functional AR signaling may influence transcriptional programs involved in cell-cycle regulation and differentiation, providing a potential biological context for this association. However, these mechanisms were not directly investigated in the present study, and our findings should therefore be interpreted as associations rather than evidence of a direct causal relationship between AR signaling and tumor proliferation.

AR expression was also significantly associated with histological grade, with lower expression observed in poorly differentiated tumors, whereas no significant association was identified with TNM stage. The association between AR expression, lower proliferation, and better histological differentiation is consistent with previous studies suggesting that AR expression is more frequently retained in tumors with a more differentiated phenotype [7]. Several meta-analyses have reported associations between AR positivity and improved disease-free and overall survival, particularly in hormone receptor-positive breast cancer [8,9,10]. However, our study did not include survival or treatment-response analyses; therefore, the prognostic implications reported in previous studies cannot be directly extrapolated to our cohort. Further longitudinal studies are required to determine whether AR expression provides independent information after adjustment for established clinicopathological factors.

The biological relevance of AR expression may be influenced by its interaction with other hormonal and oncogenic signaling pathways. In ER-positive tumors, experimental studies have suggested that AR may modulate estrogen receptor activity through effects on transcriptional regulation and chromatin binding, potentially influencing estrogen-driven proliferation and cellular differentiation. In ER-negative tumors, AR signaling may operate through distinct molecular networks involving pathways related to proliferation, metabolism, and cell survival. These mechanisms provide a biological framework for understanding the differences in AR expression observed across molecular subtypes; however, they were not directly assessed in the present study. Importantly, the presence of AR protein expression does not necessarily indicate functional dependence on androgen signaling. Consequently, AR immunohistochemical positivity should not be considered equivalent to functional AR pathway activation.

This distinction is particularly relevant in TNBC. TNBC is a heterogeneous group of tumors that includes several molecularly defined subtypes, among them the luminal androgen receptor (LAR) subtype [11,12,13]. LAR tumors are characterized by androgen-regulated and luminal-related transcriptional programs despite the absence of ER and PR expression. However, the LAR subtype is a molecularly defined entity and cannot be reliably identified on the basis of AR protein expression by immunohistochemistry alone. In the present study, AR expression was generally low in TNBC, but these findings should not be interpreted as evidence for the absence of LAR tumors. Because molecular profiling was not performed, we cannot determine the prevalence of the LAR subtype or identify functionally AR-dependent tumors within our TNBC subgroup. Future studies integrating AR immunohistochemistry with transcriptomic or other molecular approaches are needed to better characterize this population. The potential clinical relevance of AR-directed therapies has been investigated in selected AR-expressing breast cancer populations, particularly in TNBC. Clinical studies evaluating anti-androgen agents, including bicalutamide and enzalutamide, and androgen biosynthesis inhibitors such as abiraterone acetate have reported clinical activity in selected patients [15,16,17,18]. However, responses have been heterogeneous, and AR expression by immunohistochemistry alone may not be sufficient to identify tumors that are functionally dependent on AR signaling.

In the present study, we assessed AR protein expression but did not evaluate AR pathway activation, molecular subtype by gene expression profiling, or response to anti-androgen treatment. Therefore, our findings cannot be used to predict sensitivity to AR-directed therapies or to support therapeutic decisions. The therapeutic implications of AR expression in our cohort should consequently be considered exploratory. Future studies combining quantitative AR assessment with molecular characterization and clinical outcome data may help identify patients who could potentially benefit from AR-directed strategies.

Several limitations should be considered. First, the relatively small size of the TNBC subgroup (n = 31) may have reduced the statistical power of subgroup analyses and limits the generalizability of these findings to the broader TNBC population. Therefore, the inverse association between AR expression and Ki-67 observed in TNBC should be considered exploratory. The limited sample size, together with the absence of molecular profiling, also prevents definitive characterization of the LAR subtype in our cohort. Second, the absence of multivariable analyses prevents determination of whether the associations observed between AR expression and clinicopathological variables are independent of potential confounding factors. Accordingly, these relationships should be interpreted cautiously. Finally, our study did not include survival or treatment-response data, limiting assessment of the prognostic and predictive value of AR expression. Larger independent cohorts incorporating molecular profiling, longitudinal clinical outcomes, and multivariable statistical models will be necessary to validate these findings and clarify the biological, prognostic, and potential therapeutic relevance of AR expression in breast cancer.

Overall, our findings support the potential role of AR protein expression as a biological biomarker associated with breast cancer molecular and clinicopathological characteristics. However, AR immunohistochemical expression alone does not establish functional AR dependence or identify the molecularly defined LAR subtype. Further studies integrating quantitative AR assessment with molecular characterization and clinical outcome data are warranted to determine the biological and clinical relevance of AR expression across breast cancer subtypes [11,12,19,20].

## 5. Conclusions

Androgen receptor (AR) expression was detected in a high proportion of breast cancer tumors and showed substantial heterogeneity distribution across the study cohort.

Significant differences in AR expression were observed among molecular subtypes, with the highest levels identified in luminal tumors and markedly lower expression in triple-negative breast cancer (TNBC). AR expression was inversely associated with the Ki-67 proliferation index in the overall cohort and in the TNBC subgroup, suggesting an association between higher AR expression and lower tumor proliferative activity.

Furthermore, AR expression was significantly associated with histological grade, with lower expression observed in poorly differentiated tumors, whereas no significant association was identified with clinical stage.

Although AR expression was generally low in TNBC, the relatively small size of this subgroup limits the interpretation and generalizability of these findings. Overall, these findings support the potential role of AR expression as a biological biomarker associated with breast cancer molecular and clinicopathological characteristics. However, as this study evaluated AR protein expression by immunohistochemistry alone, these findings do not allow the identification of specific molecular subtypes, such as the luminal androgen receptor (LAR) subtype, or direct conclusions regarding sensitivity to anti-androgen therapies. Further studies integrating molecular profiling, larger cohorts, and clinical outcome data are warranted to clarify the biological and potential therapeutic relevance of AR expression in breast cancer.

## Figures and Tables

**Table 1:** Distribution of molecular subtypes in the cohort.

	Total N	with RA N (%)
Luminal A	143	74 (51.7)
Luminal B	166	75 (45.2)
Luminal HER2	35	20 (57.1)
HER2	29	15 (51.7)
Triple negative	53	31 (58.5%)

N: frequency; with RA N (%): frequency of each subtype with androgen receptor-positive results.

**Table 2:** Global androgen receptor (AR) expression in the study cohort.

Parameter	Value
Valid cases (n)	215
Mean ± SD	48.3 ± 31.8
Median (IQR)	53.3 (14.6–77.4)
Range	0.02–93.74

n, number; SD, standard deviation; IQR, interquartile range.

**Table 3:** Categorical androgen receptor (AR) expression according to predefined cutoffs.

AR Expression Category	Number of Cases	Percentage (%)
AR > 1%	189	87.9
AR > 10%	167	77.7

**Table 4:** AR expression according to molecular subtype.

Molecular Subtype	Median AR Expression (%)	IQR	*p*-Value	Effect Size (ε^2^)
Luminal A	69.7	38.2–83.2		
Luminal B	59.5	21.2–76.7		
HER2-positive	54.6	40.3–77.6		
Triple-negative	0.41	0.08–6.67	<0.001	
Overall			<0.001	0.244

IQR, interquartile range. AR, androgen receptor. Statistical significance was assessed using the Kruskal–Wallis test. Epsilon squared (ε^2^) was used as a measure of effect size and indicated a moderate-to-large effect.

**Table 5:** Correlation between AR expression and biological variables.

Variable	Spearman’s ρ	*p*-Value
ER	0.357	<0.001
PR	0.331	<0.001
Ki-67	−0.272	<0.001
HER2	0.021	0.764

ER, estrogen receptor; PR, progesterone receptor. Correlations were assessed using Spearman’s rank correlation coefficient.

**Table 6:** AR expression according to clinicopathological variables.

Variable	Category	Median AR Expression (%)	IQR	*p*-Value	Effect Size (ε^2^)
Histological grade	Well differentiated	61.8	36.9–80.3		
	Moderately differentiated	63.9	27.7–78.7		
	Poorly differentiated	25.5	4.5–66.3	<0.001	0.091
TNM stage	I	56.5	26.6–78.3		
	II	49.7	19.4–78.2		
	III–IV *	55.2	9.1–69.5	0.214	0.006

IQR, interquartile range. * TNM stages III and IV were combined because the number of stage IV tumors was limited, preventing meaningful statistical comparisons while preserving sufficient statistical power.

**Table 7:** Androgen receptor (AR) expression in the Triple-Negative Breast Cancer Subgroup.

Variable	Result
Number of cases (n)	31
Median AR expression (%)	0.41
IQR	0.08–6.67
Correlation between AR and Ki-67	ρ = −0.369
*p*-value	0.041

n, number; %, percentage; IQR, interquartile range.

## Data Availability

The data presented in this study are available upon request from the corresponding author. The data are not publicly available due to privacy and ethical restrictions.

## Supplementary Materials

The following supporting information can be downloaded at: https://www.mdpi.com/article/10.3390/medsci14040479/s1, Figure S1: QuPath ver. 0.6.0 Workflow for AR Quantification and Figure S1 workflow images.

## Abbreviations

The following abbreviations are used in this manuscript:

AR	Androgen Receptor
ER	Estrogen Receptor
PR	Progesterone Receptor
HER2	Human Epidermal Growth Factor Receptor 2
Ki-67	Cellular Proliferation Index
TNBC	Triple-Negative Breast Cancer
FFPE	Formalin-fixed, Paraffin-Embedded
DAB	3,3′-Diaminobenzidine
IMIB	Instituto Murciano de Investigación Biosanitaria
HCUVA	Hospital Clínico Universitario Virgen de la Arrixaca
